# Next generation sequencing uncovers multiple miRNAs associated molecular targets in gallbladder cancer patients

**DOI:** 10.1038/s41598-023-44767-3

**Published:** 2023-11-04

**Authors:** Rahul Saxena, Baskar Chakrapani, M. P. Sarath Krishnan, Amit Gupta, Sweety Gupta, Jayanta Das, Subash C. Gupta, Anissa A. Mirza, Shalinee Rao, Bela Goyal

**Affiliations:** 1 Department of Biochemistry, All India Institute of Medical Sciences, Rishikesh, Uttarakhand 249203 India; 2 Department of General Surgery, All India Institute of Medical Sciences, Rishikesh, Uttarakhand India; 3 Department of Radiation Oncology, All India Institute of Medical Sciences, Rishikesh, Uttarakhand India; 4 Department of Biochemistry, All India Institute of Medical Sciences, Guwahati, Assam India; 5 Department of Pathology and Laboratory Medicine, All India Institute of Medical Sciences, Rishikesh, Uttarakhand India

**Keywords:** Biochemistry, Biotechnology, Cancer, Diseases, Health care, Medical research, Molecular medicine, Oncology

## Abstract

Gallbladder cancer (GBC) is a lethal disease with surgical resection as the only curative treatment. However, many patients are ineligible for surgery, and current adjuvant treatments exhibit limited effectiveness. Next-generation sequencing has improved our understanding of molecular pathways in cancer, sparking interest in microRNA-based gene regulation. The aim of the study is to identify dysregulated miRNAs in GBC and investigate their potential as therapeutic tools for effective and targeted treatment strategies. GBC and control tissue samples were sequenced for miRNA expression using the Illumina HiSeq platform. Biological processes and related pathways were determined using the Panther and Gene Ontology databases. 439 significantly differentially expressed miRNAs were identified; 19 of them were upregulated and 29 were downregulated. Key enriched biological processes included immune cell apoptosis, endoplasmic reticulum (ER) overload response, and negative regulation of the androgen receptor (AR) signaling pathway. Panther analysis revealed the insulin-like growth factor (IGF)-mitogen activated protein kinases (MAPK) cascade, p38 MAPK pathway, p53 pathway, and FAS (a subgroup of the tumor necrosis factor receptor) signaling pathway as highly enriched among dysregulated miRNAs. Kirsten rat sarcoma virus (KRAS), AR, and interferon gamma (IFN-γ) pathways were identified among the key pathways potentially amenable to targeted therapy. We concluded that a combination approach involving miRNA-based interventions could enhance therapeutic outcomes. Our research emphasizes the importance of precision medicine, targeting pathways using sense and anti-sense miRNAs as potential therapies in GBC.

## Introduction

Gallbladder cancer (GBC) is the fifth most prevalent gastrointestinal malignancy worldwide and the primary cause of biliary tract neoplasm-related deaths^1,2^. According to GLOBOCAN 2020, 115,949 new cases of GBC were diagnosed, and 84,695 deaths were reported globally^3^. The etiology of gallbladder cancer (GBC) is widely considered to be complex and often encompasses a series of events including chronic inflammation, metaplasia, dysplasia, and ultimately carcinoma. Multiple genetic and epigenetic alterations have been documented at the molecular level, often implicating mutations in genes such as TP53, KRAS, and ERBB2. Furthermore, it is worth noting that dysregulation of signaling pathways such as PI3K/AKT/mTOR and MAPK might potentially play a role in tumor development and invasion. Moreover, microRNAs (miRNAs) function as key regulators of several cellular processes involved in the development, maintenance, and metastasis of gallbladder cancer (GBC). The microRNA (miRNA) has an impact on a range of cellular processes and the responsiveness of gallbladder cancer cells (GBCs) to therapeutic interventions due to its modulation of many signaling pathways^4,5^. Despite recent advancements in diagnostic techniques and therapeutic management of GBC, most patients suffer recurrent local or distant metastatic diseases with a bleak prognosis. Individuals with lesions restricted to the gallbladder mucosa usually have a 5-year survival rate of 32%, whereas those with advanced lesions have a one-year survival rate of only 10%^6^.

The sole curative treatment for GBC is radical surgical resection. Unfortunately, only a small population of GBC patients can be a candidate for surgical treatment. This is mainly due to the lack of distinct symptoms in the early stages of the disease. Even the 5-year survival after surgery with complete resections is 30–50% and 50% patients experience loco-regional recurrence^7,8^. It is therefore necessary to administer adjuvant treatment in all patients with advanced GBC, especially in those where R0 resection is not achievable. The most common adjuvant treatment for GBC patients involves gemcitabine (GEM) and cisplatin (CDDP) based chemotherapy, with or without radiotherapy^9^. While these chemotherapy options show clear benefits in other cancers, the evidence for its effectiveness in GBC is less convincing^10^. A significant percentage of chemoresistance frequently coexists with poor efficacy of chemotherapy among patients with advanced GBC. Mechanism to acquired resistance can be attributed to DNA mutations, arising from treatment, adaptive response, and natural selection of drug-resistant tumor population. Numerous novel chemoresistance targets have been uncovered recently, however there is still a need for clinical evidence about the effectiveness of GBC-specific chemotherapeutic sensitizers, and there are only a few clinical trials underway^10–12^. In addition, Folinic acid, fluorouracil and oxaliplatin (FOLFOX) is being considered as a standard-of-care in the second line setting for biliary tract cancer pre-treated patients, however overall survival benefit remains modest^13^.

Advancements in technology, such as next-generation and single-cell sequencing, have led to better understanding of intracellular events involved in cancer progression. It has paved the way for development of therapeutic agents that target crucial molecular pathways including epidermal growth factor receptors (EGFR), Vascular Endothelial Growth Factor (VEGFR), Tyrosine Kinase Inhibitor (TKI), Mitogen-activated protein kinase (MEK1/2) and BRAF (B-Raf)^14^. Nonetheless, while the response rate has improved with the use of combination treatments involving chemotherapy and targeted drugs, the overall efficacy remains suboptimal^15^. MicroRNA (miRNA), are small non-coding RNA, have garnered significant attention in recent years for their role in the regulation of gene expression. They can either upregulate or downregulate the expression of specific genes, leading to the activation or inhibition of specific signaling pathways. Besides their utility as biomarkers for diagnosis and prognosis in many diseases, including cancers, they are also being explored for the development of potential miRNA-based therapies^14,16^. Recent investigations have elucidated the potential of employing miRNA-based combination therapies, which exploit miRNAs as both sense and antisense agents’ concomitant with standard chemotherapy. These advances have been particularly evident in the treatment of ovarian, colorectal, and pancreatic cancer^17–19^. The incorporation of miRNA-based strategies in therapeutic regimens may offer a more efficacious and targeted approach to GBC treatment. Therefore, in the present study, we have attempted to identify dysregulated miRNAs in GBC that are implicated in the modulation of signaling pathways governing cancer progression. To achieve this, we employed next-generation sequencing (NGS) platform, which offers a high-throughput and comprehensive approach to elucidate the miRNA landscape and its impact on cancer-related cellular processes. This in-depth analysis aims to uncover novel miRNA targets and advance our understanding of the molecular mechanisms underlying GBC progression.

## Results

### Principal component analysis

**Figure 1:**
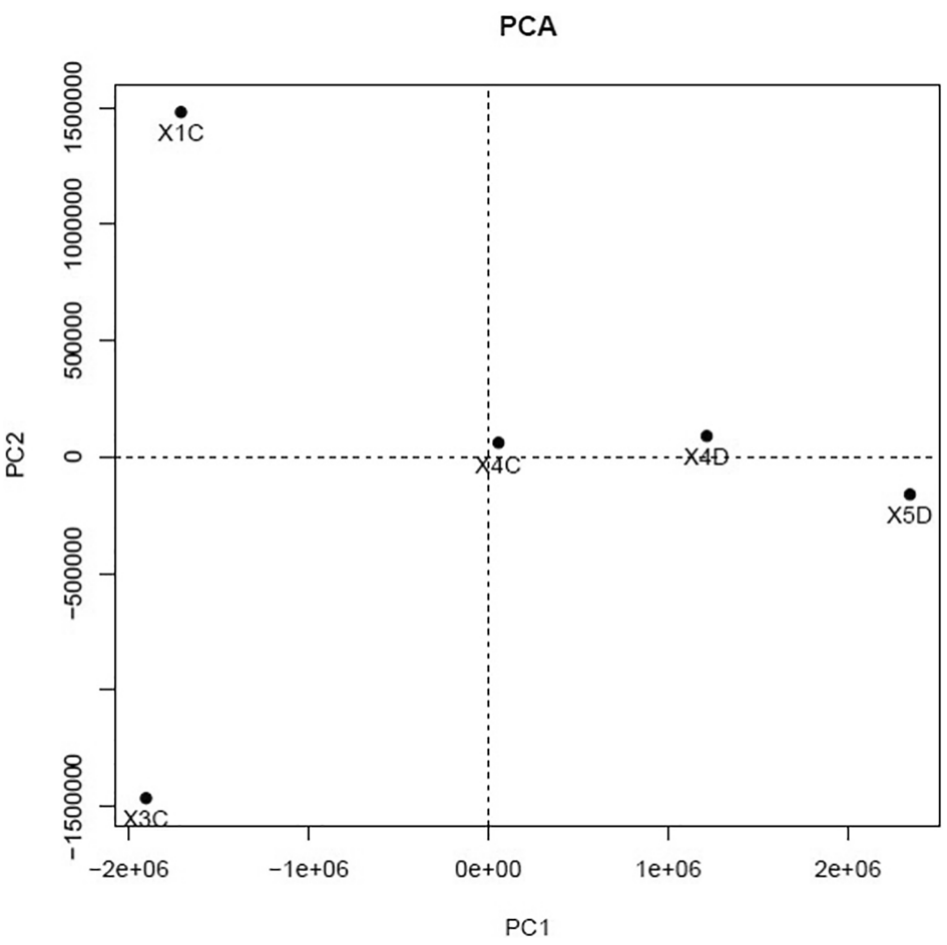
Principal component analysis of control and GBC sample. Samples X4C, X4D and X5D are close inferring similarity between samples, with X1C and X3C as dissimilar from these samples also from each other (C = Normal Sample; D = GBC Sample).

The patients included in the study had advanced stage gallbladder adenocarcinoma with stage III and stage IV disease at the time of presentation (Supplementary Table 1). The PCA of control and GBC cancer tissue sample showed that differential expressed genes value of standard samples falls in the left quadrant of the graph (top left, bottom left and middle). In contrast, the cancer sample falls on the right quadrant of the graph (Fig. 1), implicating that the GBC group and two different groups are different based on the miRNA gene expression profile.

### Upregulated and downregulated miRNAs

Among the 439 significantly (p-value < 0.05) differentially expressed miRNAs, our analysis found 19 miRNAs to be upregulated (p-value < 0.05 and Log2FC ≥ 1) and 29 miRNAs to be downregulated (p-value < 0.05 and Log2FC ≤ 1) (Table 1). These included both oncogenic and tumour suppressors miRNAs. However, we found three less known oncogenic miRNAs (mir376c5p, mir6685p and mir8875p) which were significantly upregulated in cases. The gene targets for these dysregulated miRNAs were also determined (Supplementary Table 2).

**Table 1:** List of significantly (p-value < 0.05 and Log2FC ≥ 1 and ≤ 1) expressed upregulated and downregulated miRNAs in GBC patients.

Upregulated			Downregulated		
	miRNA	log2FoldChange		miRNA	log2FoldChange
1	hsa-miR-23a-5p	30	1	hsa-miR-548aq-3p	− 21.816
2	hsa-miR-452-5p	24.53372901	2	hsa-miR-4712-5p	− 21.002
3	hsa-miR-149-5p	13.38060086	3	hsa-miR-3936	− 20.842
4	hsa-miR-1303	9.79977159	4	hsa-miR-6852-5p	− 20.577
5	hsa-miR-4732-5p	8.516018472	5	hsa-miR-378c	− 12.984
6	hsa-miR-668-5p	7.325056857	6	hsa-miR-7705	− 8.317
7	hsa-miR-486-5p	3.790573708	7	hsa-miR-4724-5p	− 7.241
8	hsa-miR-122-5p	3.215143659	8	hsa-miR-3128	− 7.178
9	hsa-miR-144-5p	2.760090004	9	hsa-miR-664b-3p	− 7.111
10	hsa-miR-766-5p	2.297476115	10	hsa-miR-3199	− 7.026
11	hsa-miR-887-5p	2.277619861	11	hsa-miR-196a-5p	− 6.780
12	hsa-miR-376c-5p	2.273984395	12	hsa-miR-561-5p	− 6.310
13	hsa-miR-432-5p	1.996713331	13	hsa-miR-1284	− 5.935
14	hsa-miR-127-5p	1.950554213	14	hsa-miR-203b-5p	− 4.507
15	hsa-miR-145-5p	1.92389115	15	hsa-miR-196b-5p	− 4.208
16	hsa-miR-154-5p	1.910857199	16	hsa-miR-203a-5p	− 4.158
17	hsa-miR-382-5p	1.876194379	17	hsa-miR-153-3p	− 3.606
18	hsa-miR-493-5p	1.723137569	18	hsa-miR-135b-5p	− 3.541
19	hsa-miR-214-5p	1.413975906	19	hsa-miR-449a	− 3.030
			20	hsa-miR-1277-5p	− 2.807
			21	hsa-miR-590-5p	− 2.573
			22	hsa-miR-499a-5p	− 2.564
			23	hsa-miR-9-5p	− 2.282
			24	hsa-miR-96-5p	− 2.258
			25	hsa-miR-32-5p	− 2.182
			26	hsa-miR-190b-5p	− 2.060
			27	hsa-miR-3613-5p	− 2.037
			28	hsa-miR-21-5p	− 1.268
			29	hsa-miR-374a-5p	− 1.208

Additionally, our analysis also revealed a total of 73 novel miRNAs in cancer samples. These miRNAs have significant Randfold p-value and no relatable material is currently available in miRBase miRNA, UCSC browser and NCBI blastn databases. MiRDeep2 assigned a temporary miRNA name, in which the first part of the ID specifies the chromosome or genome upon which miRNA gene is found, and the second part is a running number that is given to prevent duplicate IDs (Supplementary Table 3).

### Gene Ontology (GO) analysis

**Figure 2:**
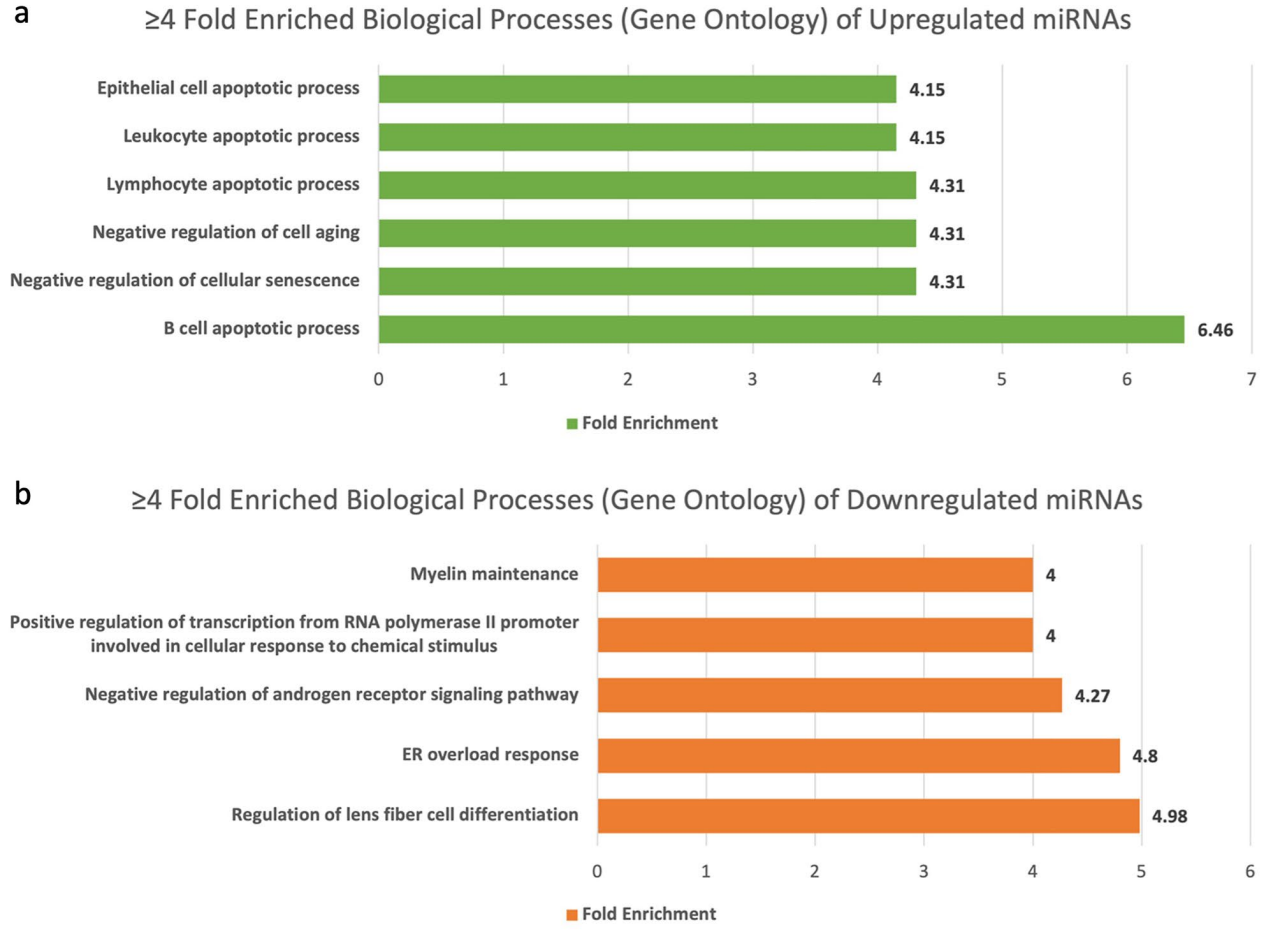
(**a**) Fold enrichment of biological processes by upregulated miRNAs through Gene Ontology database. (**b**) Fold enrichment of biological processes by downregulated miRNAs through Gene Ontology database.

In our GO analysis, we found 696 biological process to be regulated by upregulated miRNAs and 1072 to be regulated by downregulated miRNAs (Supplementary Table 4). For the purposes of visual representation we have visualised biological process which displayed more than fourfold enrichment (Fig. 2). The GO results of upregulated miRNAs revealed the highest fold enrichment with apoptosis of immune cells like B cell, lymphocytes and leukocytes, indicating role of cellular immunity in GBC. On the other hand, GO results of downregulated miRNAs showed a > 4.9-fold enrichment of regulation of lens fiber cell differentiation and high fold enrichment of endoplasmic reticulum (ER) overload response and negative regulation of androgen receptor (AR) signalling pathway.

**Figure 3:**
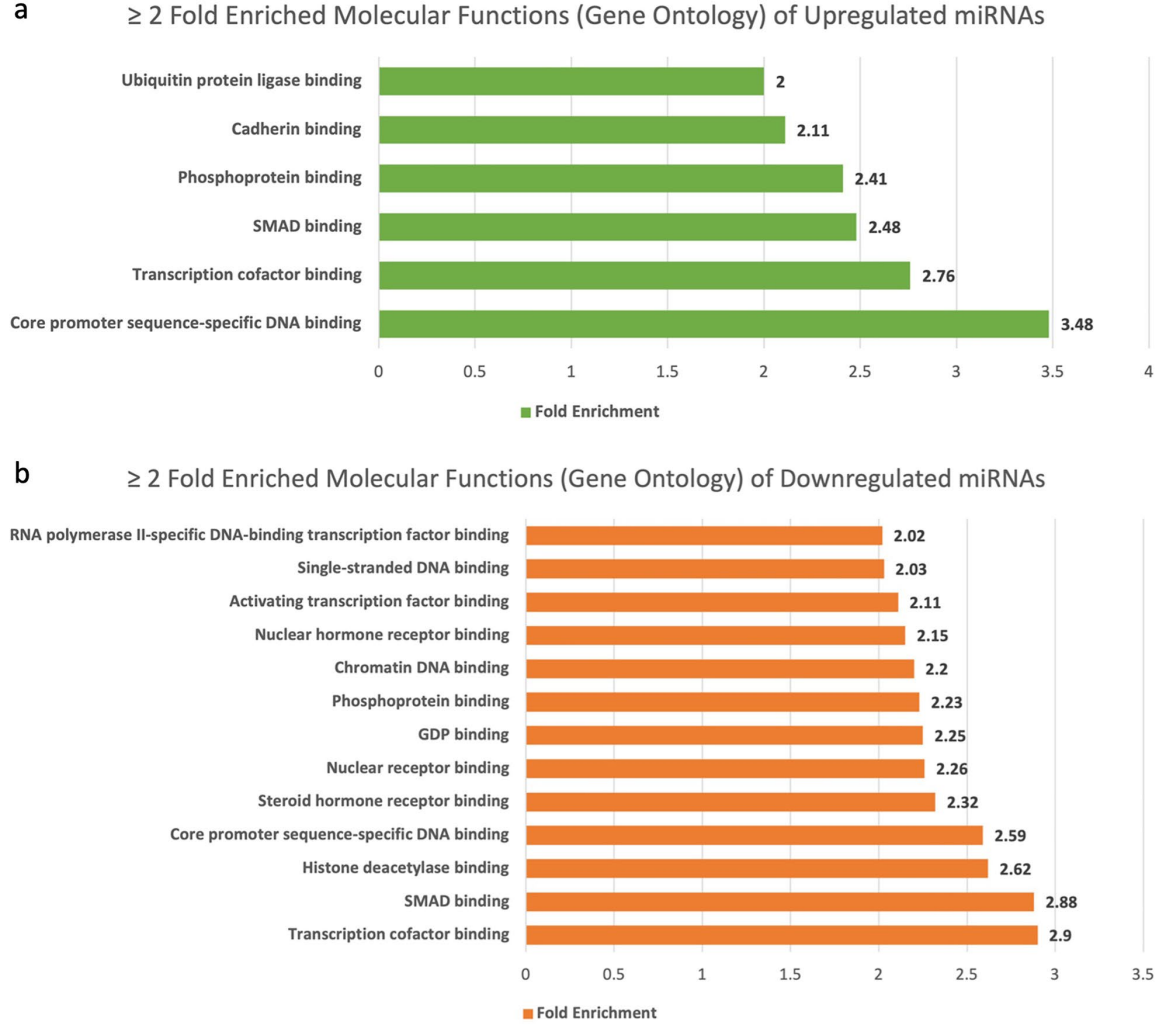
(**a**) Fold enrichment of molecular functions by upregulated miRNAs through Gene Ontology database. (**b**) Fold enrichment of molecular functions by downregulated miRNAs through Gene Ontology database.

Molecular function analysis provides insights into the specific activities performed by genes and offers valuable insights into the underlying biological processes. Our results showcase 74 molecular processes to be regulated by upregulated miRNAs, out of which core promoter sequence-specific DNA binding, transcription cofactor binding and SMAD binding were significantly enriched. In contrast, transcription cofactor binding, SMAD binding, histone deacetylase binding and core promoter sequence-specific DNA binding were found to be highly enriched among a total of 103 molecular functions regulated by downregulated miRNAs (Fig. 3) (Supplementary Table 5).

**Figure 4:**
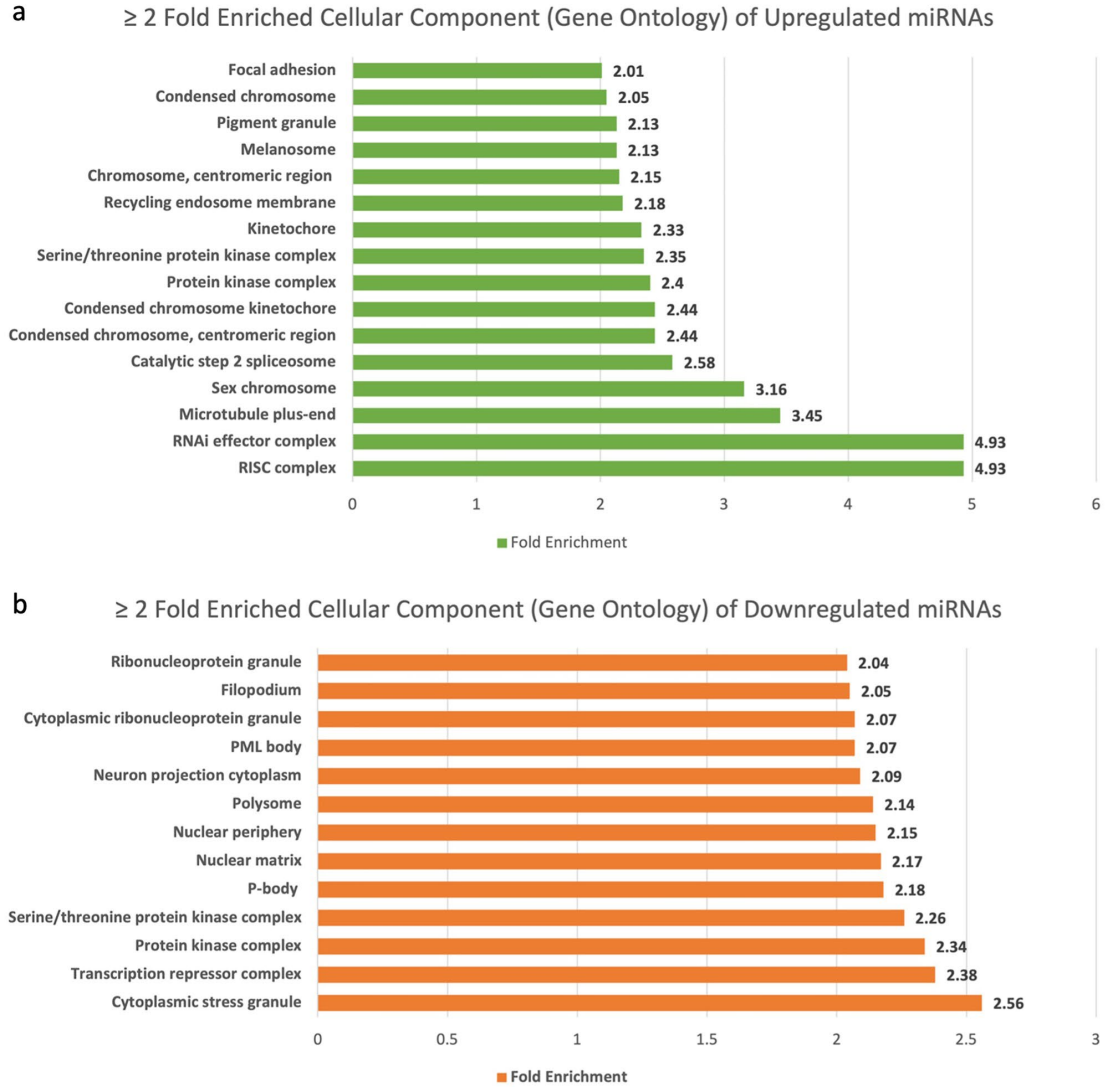
(**a**) Fold enrichment of cellular components by upregulated miRNAs through Gene Ontology database. (**b**) Fold enrichment of cellular components by downregulated miRNAs through Gene Ontology database.

Cellular component in GO analysis facilitates the understanding of subcellular organization, localization patterns of proteins, and their possible roles within specific cellular structures or complexes. We found 102 and 111 cellular components to be associated with the genes of upregulated and downregulated miRNAs respectively (Supplementary Table 6). To enhance visualization, we employed a criterion of over twofold enrichment (Fig. 4). The highest fold enrichment of upregulated miRNAs was found to be present with RNA induced silencing complex (RISC), followed by RNAi effector complex and Microtubule plus-end. On the other hand, cytoplasmic stress granule, transcription repressor complex and protein kinase complex were the highly enriched cellular components of downregulated miRNAs.

### Panther pathways analysis

**Figure 5:**
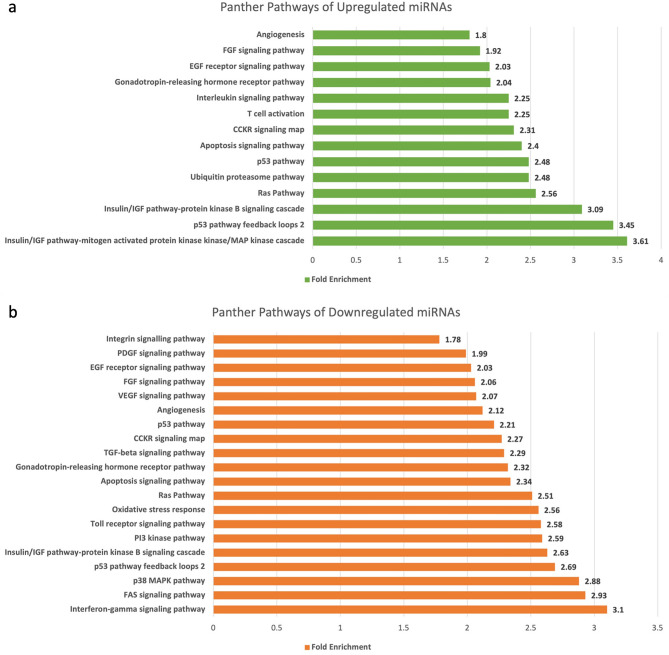
(**a**) Fold Enrichment of regulated pathways by upregulated miRNA through Panther Pathways database. (**b**) Fold Enrichment of regulated pathways by downregulated miRNA through Panther Pathways database.

The panther pathways analysis covered the majority of pathways which were also previously associated with GBC (Fig. 5). We found Insulin-Like Growth Factor (IGF) pathway, mitogen activated protein kinases (MAPK) cascade, p53 pathway and IGF pathway protein kinase B signalling cascade to be significantly enriched in gene with upregulated miRNAs. On the other hand, p38 MAPK pathway, FAS (subgroup of the tumour necrosis factor receptor) signalling pathway and interferon gamma (IFN-γ) signalling pathway were highly enriched in genes of downregulated miRNAs.

## Discussion

GBC is one of the most common biliary tract cancers and is relatively unexplored, with a dismal prognosis ranging from 19 to 62%, depending on the stage of presentation^20^. Most of the patients, however, present at an advanced stage. Surgery is the only potentially curative option, with palliative therapy in advanced stages. With the advancement of technology, a targeted approach to GBC is the need of the hour, considering the multiple facets involved in the molecular pathogenesis of GBC. miRNAs expression studies have evolved as an emerging field in understanding the pathogenesis of various cancer. miRNAs have been identified as a critical regulator of gene expression, modulating the hallmarks of cancers like cell proliferation, migration, invasion, apoptosis, radiosensitivity, chemosensitivity, and cancer stem cell phenotype. miRNAs are also being explored for their potential biomarker and therapeutic target role owing to their stability, ease of detection, and availability of synthetic oligonucleotides serving as both mimics and antisense miRNAs.

MiRNA sequencing in GBC has not been extensively explored so far. The sequencing has the advantage of not only unravelling a plethora of differentially expressed miRNAs but also has the potential to identify novel miRNAs. In the current study, we identified differentially expressed miRNAs in GBC and their targeted genes, molecular pathways, and biological processes with the help of bioinformatics tools. Our analysis found 439 significantly (p-value < 0.05) differentially expressed miRNAs. Out of this, 19 were significantly upregulated (p-value < 0.05 and Log2FC ≥ 0) and 29 were significantly downregulated (p-value < 0.05 and Log2FC ≤ 0). An attempt was then made to look for any pathways or biological processes potentially targetable for GBC.

The gene ontology and panther pathways analysis revealed immunological cell apoptosis (B Cell, lymphocyte, and monocyte), IGF pathway, and MAPK cascade were amongst the most highly upregulated biological processes and pathways. In addition, the Ras pathway, p53 pathway, and EGFR signaling pathways were also found to be upregulated. These findings suggest that cancer immunity is weakened, and cell proliferative pathways are activated in GBC, with miRNAs playing a crucial role as regulators.

In a recent study, IGF-I, IGF-II, and IGF-IR were found to be expressed at significantly higher levels in the primary tumors compared to normal gallbladder tissue. The study corroborates with our results and advocates targeting the IGF signaling pathway with therapeutic modalities like anti-IGF-IR antibodies, IGF-IR tyrosine kinase inhibitors, and IGF-IR gene silencing by antisense RNA interference^21^. MicroRNA-based therapy approaches for the IGF signaling pathway has been tried in hepatocellular carcinoma^22^ and colorectal cancer^23^ and showed promising results, hence can be further investigated for its therapeutic implications in GBC.

A recent study on genomic profiling of GBC found MAPK pathways and TGF-β, Wnt/β-catenin signalling to be the chief regulators of cancer progression in GBC^24^. Multiple studies have also reported the upregulation of MAPK pathway components in GBC tissues and cell lines. The upregulation of the MAPK pathway in GBC has been shown to promote several key oncogenic processes, including cell proliferation, survival, and invasion^25^. Furthermore, activation of the MAPK pathway has been associated with chemotherapy resistance in gastric and cholangiocarcinoma cells^26,27^. In addition, a study has revealed that both the MAPK and mTOR pathways are frequently dysregulated in GBC tissues, and targeting both pathways can inhibit cell proliferation, induce cell cycle arrest, and promote apoptosis^28^. Given the crucial role of the MAPK pathway in GBC, it is essential to explore its therapeutic potential by targeting this pathway.

The upregulation of the Ras pathway has been well-documented in cancer cells by several studies. In GBC, the expression of KRAS, a crucial component of the Ras pathway, has been reported in 3% to 30% of cases, and it is also suspected to confer resistance to anti-EGFR treatment in patients^29,30^. The overexpression of the Ras pathway can promote cancer growth by activating downstream effectors like the MAPK and PI3K/AKT pathways^31^. Recently, KRAS has emerged as a promising therapeutic target in several cancers, and miRNAs are being investigated for their potential regulatory role in this pathway^31–34^. For instance, a study by Zhou et al. demonstrated that miRNA-30a could act as a tumor suppressor by blocking the Ras/Raf/MEK/ERK signaling pathway in hepatocellular carcinoma^35^. This highlights the potential role of miRNAs as key regulators of the KRAS pathway, which not only promotes cancer progression but also confers resistance to other therapeutic modalities. Despite these findings, there is currently insufficient data on the involvement of miRNAs with KRAS pathway in GBC, and further studies are warranted to explore their therapeutic potential in this direction.

Several studies have suggested that p53 could serve as a surrogate marker for GBC^36,37^. In a previous study conducted by our group, we observed an upregulation of p53 expression in 74% of GBC patients, which was also associated with the advanced stage of cancer^38^. Notably, several studies have demonstrated that restoring p53 function via gene therapy or small molecule drugs can inhibit cancer cell growth and trigger apoptosis in various cancers, such as prostate and cervical cancers^39,40^. MicroRNAs are crucial components of the p53 signalling pathway, acting upstream and downstream of this transcription factor. A study on colorectal cancer showed that miRNA-16 could suppress cell growth by regulating the p53/survivin signalling pathway^41^. Given the essential role played by the p53 pathway in several cancers, including GBC, it is imperative to investigate the potential therapeutic applications of miRNA-based strategies targeting this pathway.

On the other hand, ER overload response and negative regulation of the androgen receptor signalling pathway were among the processes affected by downregulated miRNAs indicating an increased ER stress and increased signalling of AR. Multiple studies have suggested a link between ER stress and cancer, particularly the involvement of the unfolded protein response (UPR)^42^. ER stress could enable cancer cells to return to homeostasis while promoting the growth and survival of the tumour in the surrounding tissue^43^. Thus downregulation of a response against ER overload helps promote gallbladder carcinogenesis. Androgen receptor signalling have been highly studied in breast cancer and prostate cancer progression^44–46^. Targeted therapies based on AR are already in clinical practice in prostate cancer. Contrary to this, a single study was found on the risk of biliary tract cancer and gallstones development with AR CAG repeat length and concluded no association was present^47^. One plausible explanation for this could be that AR signalling may be affected at epigenetic level by miRNAs as observed in current study rather than genetic level which needs to be explored further. Therefore, our results on the downregulation of negative regulation of the androgen receptor suggests the high activity of AR during the progression of cancer that may be due to effect of miRNA regulation at mRNA level. Moreover, our panther analysis revealed downregulation of IFN-γ signalling pathway. The downregulation of the IFN-γ signalling pathway is associated with immune evasion and cancer progression in colorectal cancer, similar mechanism of action has been ruminated for GBC^48^. Decreased expression of IFNGR1 and STAT1 has demonstrated to decrease sensitivity of cancer cells to the immune response mediated by IFN-γ^48–50^. Interestingly, studies have reported a correlation between miRNAs and STAT. A study found that miRNA-23 targets STAT and suppresses its expression, promoting prostate cancer progression^51^. These findings suggest that miRNAs and STAT may play a complex role in the development and progression of GBC. Overall, further research is needed to fully understand the role of miRNAs and STAT1 in GBC and to explore their potential as therapeutic targets for this disease.

The current study attempts to provide a proof of principle that miRNAs may be the key player regulating potential targetable pathways dysregulated in GBC. Although the sample size is too small to support a conclusive analysis and requires additional validation, it may indicate potential areas for future research. Although the therapeutic potential of sense and antisense miRNAs has been studied in hepatocellular carcinoma and cholangiocarcinoma, research in GBC is still in its nascent stage^18,52,53^. Considering the similarities between hepatobiliary cancers and GBC, investigating the potential of miRNA-based therapies in GBC may open up new treatment avenues for this highly aggressive cancer. Recent studies have highlighted the potential of sense and anti-sense miRNAs therapy in sensitizing GBC cells to chemotherapy, leading to improved treatment outcomes. Mittal et al. showed that miRNA-205 mimics significantly increased the pancreatic cancer cells sensitivity to gemcitabine, a widely used chemotherapy drug in cancers^19^. Similarly, liposomal miRNA-15a and miRNA-16, sensitized chemo-resistant orthotopic OvCa mouse model to cisplatin, another commonly used chemotherapy drug^17^. Furthermore, combination therapy of sense and anti-sense miRNAs with chemotherapy has shown promise in preclinical studies. A study reported that co-delivery of miRNA-100 and anti-miRNA-21 with temozolomide using nanoparticles significantly enhanced the survival in glioblastoma xenograft-bearing mice^54^. In our study, miRNA-23a is found to be the most upregulated miRNA and previous studies have also shown that miRNA-23a acts through MAPK and JAK/STAT pathways in prostate cancer^51^. miRNA-23a is also seen to downregulate the expression of interferon regulatory factor-1 in hepatocellular carcinoma cells^55^. The clinical trials based on TKI are already under investigation in GBC. Based on the results, alternative approach for poor responder subgroup may be combination of targeted drug and miRNA-based therapy. The therapy for kinase inhibitors in GBC is already under trial. Hence, these preclinical studies indicate that the use of sense and anti-sense miRNAs therapy in combination with standard chemotherapy could be a promising approach for GBC treatment. However, more investigation is necessary to assess both the safety and effectiveness of this strategy in clinical studies.

## Materials and methods

### Patient sample collection and processing

The study was approved by All India Institute of Medical Sciences, Rishikesh institutional ethical board (177/IEC/IM/2019). After obtaining informed written consent from the GBC patients and healthy controls, the cancer tissue (2 in number) was obtained from excised gallbladder during surgery and stored in RNA later at − 20 °C until the experiments were performed. All methods and procedures conducted during the course of this study were performed in accordance with applicable ethical standards, guidelines, and regulations. Gallbladder tissue excised as a part of specimen in surgery, e.g., Whipple’s procedure, right hepatic resection, etc., for normal non-dysplastic gallbladder (3 in number) was taken as a control. The miRNeasy Mini Kit (Qiagen, Cat#217004) was used to extract RNA from these specimens, and the Qubit RNA BR Assay (Invitrogen, Cat# Q10211) was used to quantify all samples. QIAxpert was used to examine RNA quality, and Tape Station was used to analyse RNA integrity using RNA screen Tapes (Agilent, Cat# 5067–5576).

### RNA library prep and miRNA sequencing

NEB Next Multiplex Small RNA Library Prep Kit was utilized to prepare the libraries. First, 200 ng of total RNA was denatured under elevated temperatures. The denatured RNA was then ligated to a 3ʹ SR adapter, followed by SR-RT primer annealing to convert the free single-stranded adapter to the double-stranded DNA molecule. Reverse transcriptase was used to copy the 3ʹ SR adapter-ligated molecules into first-strand cDNA. Following that, the adapter-ligated products were purified and enriched using the thermal conditions provided here: initial denaturation at 94 °C for 30 s; 15 cycles at 94 °C for 15 s, 62 °C for 30 s, 70 °C for 15 s; final extension at 70 °C for 5 min. After that, PCR products were purified, and fragment size distribution was examined on Tape Station using D1000 DNA Screen Tapes (Agilent, Cat# 5067–5582), accompanied by size selection on 4% E-gels. The Qubit High Sensitivity Assay (Invitrogen, Cat# Q32852) was used to quantify the libraries that had been produced. Before cluster amplification on the Illumina flow cell, the resulting libraries were merged and reduced to the final optimum loading percentage. The cluster flow cell is then put into the HiSeq 2500 instrument to generate 25 M 50 bp single-end reads.

### Pre-processing of raw data and analysis

To create and de-multiplex raw fastq sequences, the CASAVA v1.8 pipeline was used. The utility Fastq was used to assess the read quality using the settings from the fastq file (Version 0.11.9). The estimated average composition per reading, GC concentration in the reads, PCR amplification problem, over-represented motifs, and adapters were all examined for removal. Cutadapt was used for the adapter trimming (ver 1.16). Reads were sorted for lengths of 17–30 bp. Contamination was also removed utilizing Bowtie2 (version-2.2.4), which removed structural RNA contamination comprising ribosomal RNAs and transfer RNA sequences.

### Principal component analysis (PCA)

The PCA analysis was performed to identify any significant difference between the GBC tissue samples and controls. Filtered reads were mapped to the known miRBase mature and precursor using miRDeep2.0.0.8. The miRDeep2 employs Bowtie (version-0.33) for mapping the reads to mature and precursor sequences. The known miRNA was filtered for a probability score greater than zero and a Randfold p-value equal to YES. The aligned reads were used to estimate the miRNA genes’ expression using mirDeep2. MirDeep2 was also used to predict novel miRNAs, and the detected novel miRNAs were filtered for Randfold YES filtering, and Star read counts higher than or equal to 1. The star read count filter greater than or equal to 1 indicates the presence of star sequences. As detection is a crucial requirement for miRNA validation, this validates the legitimacy of the relevant miRNAs. Depth of reading coverage was assessed based on known miRNA gene loci. The normalized read count value was used to represent the PCA plot.

### Differential expression analysis

Differential expression analysis was performed to identify significant changes in various mRNA expressions. Two different methods for different comparisons were used, for pairwise comparison, Log2 standard deviation method was used, and for group comparison, DESeq2 was used. For paired comparisons, the ratio of normalized read counts was taken as the fold change. A distribution of these log2 (fold change) values was found to be normally distributed. Those genes determined to be two standard deviations off the mean, i.e. [mean + 2 × standard deviation] and [mean − 2 × standard deviation], were considered statistically significant. For group comparison, significant miRNA was filtered based on p < 0.05 and, Log2foldchange > 1 was taken as upregulated miRNA, Log2foldchange < 1 values were taken as downregulated miRNA. Target Prediction for known miRNAs was made using mirwalk 2.0, and for the novel one, the targets were predicted using miranda (ver 3.3).

### Analysis of Gene Ontology terms and Panther pathways

Gene Ontology (GO) annotation and Panther pathway information for target genes of differentially expressed miRNAs were retrieved using their respected databases. The differentially regulated miRNAs target genes were analysed to identify the biological process, molecular function, cellular compartment and pathway disease association affected by GBC. The fold enrichment for all these datasets was evaluated by comparing the background occurrence of total genes assigned to that term to the sample frequency, which represents the number of genes inputted that are annotated to that term. Disproportionate representation was defined as the positive fold enrichment value when a term was disproportionately represented compared to the background. Only data with a p-value of < 0.05 assessed by the “Mann Whitney” U-test were selected and given from lowest to highest fold enrichment. The p-value is the probability of finding (n) genes annotated to a specific term in a genomic sequence. Only ≥ twofold and higher cut-off enriched biological processes, molecular function, cellular components, and pathways were chosen for depiction. The results were entered into the revigo database for analysis, and graphs were plotted in excel.

### Ethical approval

The All India Institute of Medical Sciences, Rishikesh’s institutional ethical board approved all the procedures (177/IEC/IM/2019).

### Consent to participate

Written informed consent was obtained from all participants.

## Conclusion

Our study is an additional attempt that employs miRNA sequencing to uncover pathways modulated by miRNAs, which could be targeted for therapy in GBC. Given the resistance to treatment observed in GBC, a combinatorial approach incorporating miRNA-based interventions might offer an alternative strategy to enhance therapeutic outcomes. This study highlights the necessity for precision medicine that targets potential pathways through not only receptor inhibitors or antibodies but also by investigating miRNAs as a promising therapeutic modality.

## Supplementary Information

Supplementary Tables.

## Data Availability

The RNA sequencing files generated during this research have been deposited in the NCBI Sequence Read Archive (BioProject: PRJNA1227735, accession: SRX27791349 – SRX27791353). All other data generated or analysed during this study are included in this published article (and its Supplementary Information files).

## Abbreviations

GBC: Gallbladder cancer
miRNA: MicroRNA
ER: Endoplasmic reticulum
AR: Androgen receptor
IGF: Insulin-like growth factor
MAPK: Mitogen activated protein kinases
KRAS: Kirsten rat sarcoma virus
IFN-γ: Interferon gamma
SMAD: Suppressor of mothers against decapentaplegic
GDP: Guanosine diphosphate
RISC: RNA-induced silencing complex
RNAi: RNA interference
P-body: Processing body
PML: Promyelocytic leukaemia body
IGF: Insulin-like growth factor
MAP: Mitogen activated protein
FAS: Fas cell surface death receptor
PI3 kinase: Phosphoinositide 3-kinase
TGF-beta: Transforming growth factor-beta
CCKR: Cholecystokinin receptor
VEGF: Vascular endothelial growth factor
FGF: Fibroblast growth factor
EGF: Epidermal growth factor
PDGF: Platelet-derived growth factor
