# Author Correction: Next generation sequencing uncovers multiple miRNAs associated molecular targets in gallbladder cancer patients

**DOI:** 10.1038/s41598-025-98169-8

**Published:** 2025-04-16

**Authors:** Rahul Saxena, Baskar Chakrapani, M. P. Sarath Krishnan, Amit Gupta, Sweety Gupta, Jayanta Das, Subash C. Gupta, Anissa A. Mirza, Shalinee Rao, Bela Goyal

**Affiliations:** 1 Department of Biochemistry, All India Institute of Medical Sciences, Rishikesh, Uttarakhand 249203 India; 2 Department of General Surgery, All India Institute of Medical Sciences, Rishikesh, Uttarakhand India; 3 Department of Radiation Oncology, All India Institute of Medical Sciences, Rishikesh, Uttarakhand India; 4 Department of Biochemistry, All India Institute of Medical Sciences, Guwahati, Assam India; 5 Department of Pathology and Laboratory Medicine, All India Institute of Medical Sciences, Rishikesh, Uttarakhand India

Correction to: *Scientific Reports* 10.1038/s41598-023-44767-3, published online 04 November 2023

The original version of this Article contained errors. In the Discussion, the Article omitted an acknowledgement of limitations of the study.

“The current study attempts to provide a proof of principle that miRNAs may be the key player regulating potential targetable pathways dysregulated in GBC.”

now reads:

“The current study attempts to provide a proof of principle that miRNAs may be the key player regulating potential targetable pathways dysregulated in GBC. Although the sample size is too small to support a conclusive analysis and requires additional validation, it may indicate potential areas for future research.”

In addition, owing to issues in deposition of the original sequencing data, the Data availability section was incomplete.

“All data generated or analysed during this study are included in this published article (and its Supplementary Information files).”

now reads:

“The RNA sequencing files generated during this research have been deposited in the NCBI Sequence Read Archive (BioProject: PRJNA1227735, accession: SRX27791349 – SRX27791353). All other data generated or analysed during this study are included in this published article (and its Supplementary Information files).”

The original Article has been corrected.

